# Pott's disease: a case of *Mycobacterium xenopi* infection of the spine

**DOI:** 10.3402/jchimp.v2i4.20150

**Published:** 2013-01-07

**Authors:** Majd Alfreijat, Chiagozie Ononiwu, Carlton Sexton

**Affiliations:** 1 Department of Internal Medicine, MedStar Union Memorial Hospital, Baltimore, MD, USA; 2 Department of Radiology, MedStar Union Memorial Hospital, Baltimore, MD, USA

**Keywords:** pott's disease, Mycobacterium xenopi, Nasca culture

## Abstract

Pott's disease is an infection of the spine with *Mycobacterium tuberculosis* that causes destruction of the spine elements resulting in progressive kyphosis. We are describing a rare case of Pott's disease where *Mycobacterium xenopi* was the inculpated organism.

A 44-year-old Caucasian male with a history of HIV, Kaposi's sarcoma (not on HAART), coronary artery disease status post coronary artery bypass graft, and HTN presented to the ED with complaints of chest pain of 1 day duration, as well as back pain. A physical exam was significant for point tenderness in the lower thoracic spine. Laboratory studies showed normal blood counts and electrolytes, with slight troponin and transaminase elevations. CD4 count was 674 and HIV viral load was 52,808. Chest CT revealed osteomyelitis and discitis at T9–T10 level with a right paravertebral phlegmon.

Review of patient records from an outside hospital showed that, 5 months prior to this, a lytic lesion at T9–T10 level had been identified on imaging studies. Subsequent CT showed collections within the T9 and T10 vertebral bodies, with destruction of the intervening disc, as well as paravertebral fluid collections consistent with discitis and osteomyelitis (Fig. 1).

**Fig. 1:**
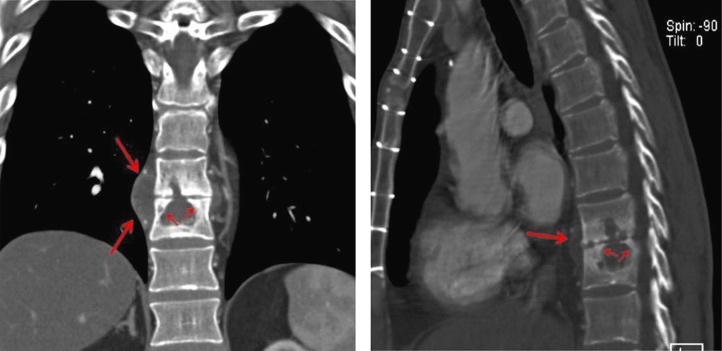
A coronal (left) and a lateral (right) reformation from the CT scan better demonstrate the paraspinal swelling (long arrows) and the collapsed disc, the lytic defects in the vertebra from tuberculous osteomyelitis, and the healing sclerosis postantibiotic treatment (short arrows).

The patient underwent a CT-guided biopsy that revealed a thick yellowish fluid that grew *Mycobacterium xenopi*. Quantiferon test for TB was negative. The patient was restarted on HAART and also placed on clarithromycin, ethambutol, moxifloxacin, and rifabutin. Acute coronary syndrome was ruled out on this admission, and the patient signed himself out against medical advice the following day.

## Definition and name origin

Pott's disease is defined as an infection of the spine with *Mycobacterium tuberculosis* that causes destruction of its elements, including the disc space and the vertebral bodies, resulting in progressive kyphosis (1, 2). It was first described in 1779 by an English surgeon named Percivall Pott (1714–1788). He was a renowned surgeon and was once described by Sir James Paget as the ‘Complete Surgeon’. Not confined to the description of the skeletal TB, his writings on the management of head injuries, and subperiosteal abscesses associated with osteomyelitis, were of great influence (3, 4).

## Pott's disease in ancient Americas

**Fig. 2:**
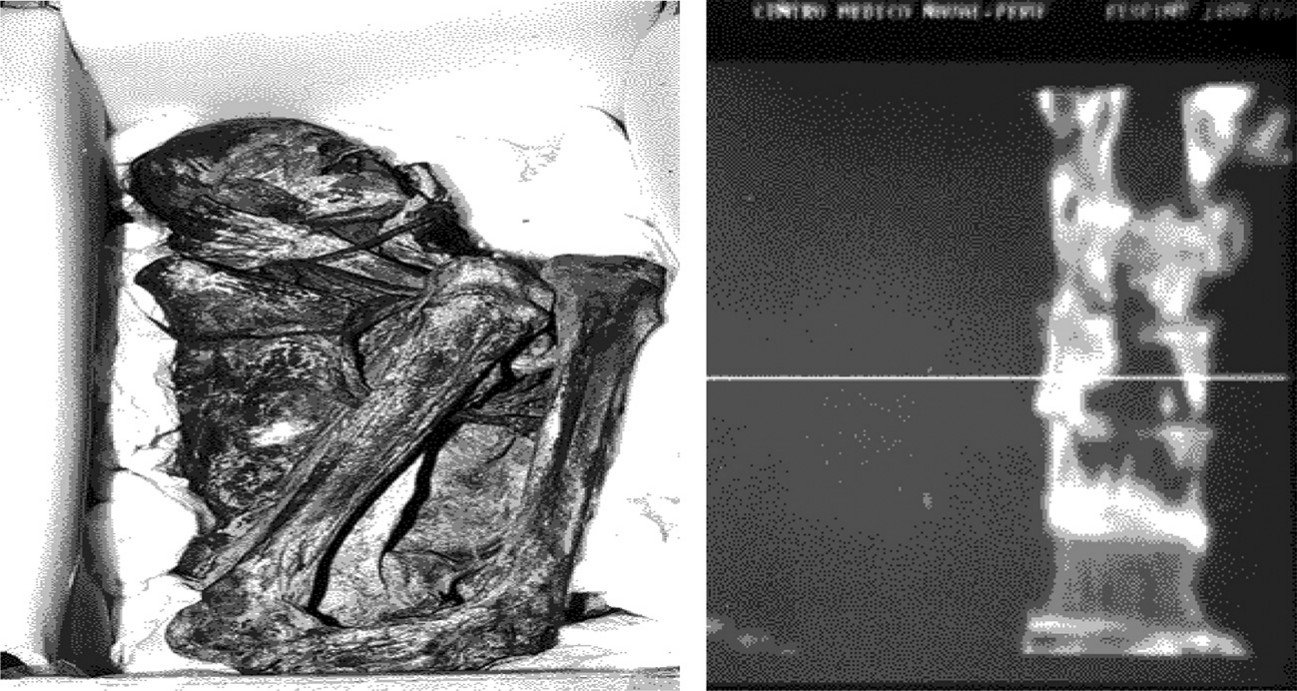
An adult male mummy from Nasca in the National Museum of Lima (left), with coronal CT scan of his spine (right) showing an osteolytic lesion involving T10.

Using non-destructive autopsy, radiological assessments, and DNA analysis, Pott's disease has been diagnosed in 5 out of 1000 mummies studied from the Nasca society – an archeological culture that prospered beside the river valleys of the Rio Grande de Nasca by the southern coast of Peru between 100 and 1000 AD. With cases concentrated in the 900 AD period, it might have reached pandemic proportions, suggesting a prevalence of tuberculosis in 10–25% of the population in this studied era. Gross and radiologic stigmata include kyphosis, osteolytic lesions, pleuro-pulmonary adherence, and collapse of the intervertebral discs. Evidence of cold abscesses was also found in the studied mummies (Fig. 2). While the origin of tuberculosis in the Americas remains debated, these findings prove the existence of the disease in pre-Columbian times, serving as the precedent, and potentially, the source for the European outbreak in the 17th century (5).

## Discussion

**Fig. 3:**
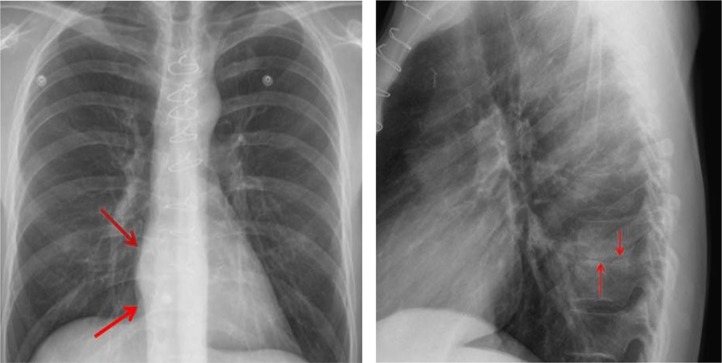
A frontal view of the chest (left) demonstrates subtle evidence of right greater than left paraspinal soft tissue swelling around the low thoracic spine (arrows). Note that the lungs are clear of any evidence of active TB. A lateral view of the chest (right) shows loss of disc height at T9–10 (arrows) and subtle sclerosis.

On both the chest X-ray and chest CT, our patient manifests imaging findings typical of infectious spondylitis, either pyogenic or tuberculous. The organism spreads hematogenously to the spine, lodging in end vessels usually anteriorly in the vertebral body, beneath the endplate (6). Chest X-rays can be the first imaging tip-off to spinal infection, when the thoracic spine is involved. In the lateral view, loss of disc height and endplate destruction may be visible (Fig. 3).

Using MRI, infection in the disc, bone, and in soft tissues is seen to be manifested by increased T2 signal and decreased T1 signal. Unlike pyogenic discitis where a ‘bright’ disc is an early sign, TB discitis may not alter signal within the disc, the ‘disc sparing’ phenomenon. MRI mainly allows for a better view of infection that may have spread into the spinal canal, outside the dura, and that may be compressing the spinal cord (7).

The differential diagnosis for destructive lesions of the spine with paraspinal soft tissue swelling also includes metastatic malignancy, primary bone tumor, and lymphoma. Other more unusual inflammatory conditions that can mimic TB of the spine include sarcoidosis, ecchinococcosis, brucellosis, and actinomycosis (8).

While mycobacterial spinal infection is predominantly caused by *M. tuberculosis* (9), there have been very few cases in the literature where other mycobacteria have been inculpated. *Mycobacterium xenopi*, which our patient had, was first isolated in 1959 from skin lesions on an adult female *Xenopus laevus* (African clawed frog) (10). Since 1965, when the first case of *M. xenopi* infection in humans was published (11), only eight cases of *M. xenopi* involving the spine have been reported (12–19).

The optimal treatment for *M. Xenopi* is yet to be identified. However, a recent study on mice showed significant bactericidal effect with ethambutol/rifampin combination with either clarithromycin or moxifloxacin (20).
